# The Perils of Perfection: Navigating the Ripple Effects of Organizational Perfectionism on Employee Misbehavior through Job Insecurity and the Buffering Role of AI Learning Self-Efficacy

**DOI:** 10.3390/bs14100937

**Published:** 2024-10-12

**Authors:** Byung-Jik Kim, Hyun-Joo Oh, Min-Jik Kim, Dong-gwi Lee

**Affiliations:** 1 Department of Psychology, Yonsei University, Seoul 03722, Republic of Korea; kimbj8212@ulsan.ac.kr (B.-J.K.); hyunjoo6768@yonsei.ac.kr (H.-J.O.); 2 College of Business, University of Ulsan, Ulsan 44610, Republic of Korea; 3 School of Industrial Management, Korea University of Technology and Education, Cheonan 31253, Republic of Korea

**Keywords:** organizationally prescribed perfectionism, self-efficacy in artificial intelligence learning, job insecurity, counterproductive work behavior, moderated mediation model

## Abstract

This study investigates the complex interplay between organizationally prescribed perfectionism (OPP), job insecurity, counterproductive work behavior (CWB), and self-efficacy in learning artificial intelligence (AI) in the context of modern organizations. Based on several theories, the current research suggests and tests a moderated mediation model. Using a three-wave time-lagged design with data collected from 412 workers across various South Korean corporations, we examine how OPP influences CWB both directly and indirectly through job insecurity, and how self-efficacy in AI learning moderates the OPP–job insecurity link. Our results show that OPP is positively linked to CWB, and this association is partially mediated by job insecurity. Moreover, AI learning self-efficacy functions as a moderator in the OPP–job insecurity link, such that the positive link is weaker for members with higher levels of AI learning self-efficacy. These findings extend our understanding of perfectionism in organizational settings and highlight the role of technological self-efficacy in mitigating the negative impacts of perfectionist cultures. This research may contribute to the literature on perfectionism, CWB, and technological adaptation at work, and has important implications for managing high-performance cultures in the period of rapid technological advancement.

## 1. Introduction

In today’s hypercompetitive and rapidly evolving global business landscape, organizations are increasingly adopting exceptionally high-performance standards as a means to maintain and enhance their competitive advantage [1,2]. This intensification of organizational expectations has given rise to the concept of organizationally prescribed perfectionism (OPP), a phenomenon that extends the well-established construct of socially prescribed perfectionism into the specific context of organizational dynamics. OPP reflects the shared perception among employees that their organization demands flawless performance and holds them to unrealistically high standards [3,4]. The emergence of OPP can be attributed to several interrelated factors shaping the modern business environment. First, the acceleration of technological advances, particularly in artificial intelligence (AI) and automation, has raised the bar for human performance, forcing organizations to strive for ever-higher levels of efficiency and innovation [5]. Second, the intensification of global competition has narrowed the margin for error, pushing organizations to cultivate a culture of perfection to maintain their market position [6]. Third, the increased transparency brought about by digital communication and social media has amplified the potential repercussions of organizational missteps, further fueling the drive towards perfectionism [7].

The pervasive influence of OPP on employees’ perceptions, attitudes, and behaviors warrants thorough investigation, especially in competitive and demanding work environments. As organizations strive for excellence, the pressure placed on employees can lead to a cascade of psychological and behavioral outcomes with far-reaching implications. These outcomes not only affect individual well-being but can also have a significant impact on organizational effectiveness, innovation capacity, and long-term sustainability [8]. Furthermore, the potential downside of OPP cannot be overlooked. While the pursuit of excellence can drive organizational success, excessive or misaligned perfectionist expectations can lead to unintended negative consequences. These may include increased employee burnout, reduced job satisfaction, heightened turnover intentions, and even ethical compromises in the pursuit of unrealistic goals [2,3]. Therefore, examining the multifaceted effects of OPP is essential not only for optimizing performance but also for ensuring sustainable organizational practices and employee well-being in today’s high-pressure business world [9,10].

Despite the growing recognition of the influence of perfectionism in several social contexts, there are significant research gaps in the existing body of literature concerning its manifestation in organizational settings. First, while scholars have extensively examined the influence of socially prescribed perfectionism on individual behaviors [1,10], the specific concept of OPP remains understudied. Given that organizations represent a critical social context for many individuals, investigating the influence of perfectionism within this specific domain is of paramount importance [2]. In particular, exploring how OPP affects employees’ work behaviors, especially negative behaviors such as counterproductive work behavior (CWB), is crucial. CWB, which includes actions that have a detrimental impact on the organization or its members, can have severe consequences for organizational performance and workplace harmony [11]. Thus, understanding the relationship between OPP and CWB can provide valuable insights for mitigating potentially detrimental workplace behaviors.

Second, existing works have not adequately investigated the mediating mechanisms (mediators) and contingent factors (moderators) in the OPP-CWB link [2]. Investigating these mediating and moderating variables is essential to developing a comprehensive knowledge of this relationship. Specifically, employees’ job insecurity may be a critical mediating factor that can explain why OPP significantly influences CWB. Job insecurity, defined as the subjective perception of a potential threat to the ongoing and stable nature of one’s employment, has been recognized as a significant stressor at work [12,13]. Examining job insecurity as a potential mediator can shed light on the psychological mechanisms through which perfectionist organizational expectations are translated into counterproductive behaviors.

Lastly, the buffering role of self-efficacy, particularly in the field of AI, has been under-researched in relation to OPP and its outcomes. In an era where AI is rapidly transforming work processes across industries, an individual member’s faith in his or her capacity to learn and effectively utilize AI technologies has become increasingly important [14,15,16,17]. Self-efficacy in AI learning represents a critical personal resource that can influence how employees respond to perfectionist organizational demands in technologically advanced work environments [18,19,20,21]. Investigating this specific form of self-efficacy as a potential moderator can provide insights into variables that may reduce the detrimental influences of OPP on members’ attitudes and behaviors.

To address these research gaps, the present study adopts a comprehensive approach based on solid theoretical foundations. Drawing on the Conservation of Resources (COR) theory, the Effort–Reward Imbalance (ERI) model, Social Exchange Theory, Psychological Contract Theory, Affective Events Theory, Social Cognitive Theory, and the Context–Attitudes–Behavior framework, we propose an integrated framework to delve into the link between OPP, job insecurity, CWB, and self-efficacy in AI learning. By investigating these relationships, the current research seeks to contribute to a deepened knowledge of the influence of perfectionist organizational cultures in the context of technological advancement and workplace dynamics.

The primary objectives of this study are as follows:

1. To examine the direct relationship between OPP and CWB in the context of modern organizations.
2. To investigate the mediating role of job insecurity in the relationship between OPP and CWB.
3. To assess the moderating effect of self-efficacy in learning AI on the relationship between OPP and job insecurity.
4. To test a comprehensive moderated mediation model that integrates these relationships and provides a nuanced understanding of how perfectionist organizational cultures influence employee behavior in the era of AI advancement.

The remainder of this paper is structured as follows: First, we present our theoretical framework and develop our hypotheses, drawing on relevant literature in organizational behavior, perfectionism, and technological adaptation. Next, we describe our methodology, including sample characteristics, data collection procedures, and analytical approaches. We then present our findings, detailing the results of our hypothesis tests and additional analyses. Finally, we discuss the theoretical and practical implications of our findings, acknowledge the limitations of our study, and suggest directions for future research. Through this structure, we aim to provide a comprehensive examination of the relationships between organizationally prescribed perfectionism, job insecurity, counterproductive work behavior, and self-efficacy in AI learning.

This study offers several important contributions: (1) extending the concept of socially prescribed perfectionism to the organizational context, (2) illuminating the mediating effect of job insecurity in the OPP-CWB link, (3) examining the moderating role of self-efficacy in learning AI, and (4) providing a holistic framework for understanding the interplay between organizational expectations, employee perceptions, and behavioral outcomes in technologically advanced workplaces. These contributions advance both theoretical knowledge and practical management strategies in an era of rapid technological development and intense organizational pressures. Taken together, these contributions not only advance theoretical knowledge but also offer practical implications for managing the potential negative consequences of perfectionist organizational cultures in an era of rapid technological development.

## 2. Theory and Hypotheses

### 2.1. OPP and Employee CWB

We propose that OPP can increase CWB. OPP extends the notion of socially prescribed perfectionism into the organizational domain. In this framework, the organizations assume the role of the “social other” by holding employees to unattainably high standards and expectations [1,22]. OPP embodies the belief that one’s organization demands perfection and posits that adherence to these stringent standards is essential for acceptance and success in the workplace [2,8,23]. Within the corporate environment, perfectionism is influenced by various variables, including organizational cultures, leadership approaches, and performance management systems [2,4]. Organizations that demand impeccable performance, have a low tolerance for error, and cultivate a highly competitive atmosphere may inadvertently encourage OPP among their employees [1,9]. Existing research has shown that OPP can harm employee well-being and performance. Employees who believe that their organization expects perfection may suffer from increased stress, burnout, and job dissatisfaction [2,4]. The relentless pursuit of meeting unachievable standards often creates fear of failure, encourages procrastination, and hinders the willingness to take risks or explore innovative methods [2,9,10].

CWB encompasses a range of deliberate employee activities that inflict damage or are specifically designed to inflict damage on organizations and their stakeholders [11,24]. This concept has garnered significant attention as a result of its significant impact on organizational functioning, productivity, and overall workplace climate [25,26,27]. These behaviors can range from minor acts such as taking excessive breaks or spreading rumors to more serious acts such as theft, sabotage, or physical aggression [11,28]. Previous work has demonstrated that the consequences of CWB are far-reaching and predominantly negative. At the organizational level, CWB can lead to significant financial losses, reduced productivity, and reputational damage [29,30]. At the individual level, targets of CWB may experience reduced job satisfaction, increased stress, and reduced physical and psychological well-being [26,27,31].

The OPP-CWB link is comprehensively explained by integrating three complementary theoretical frameworks: the COR theory, the ERI model, and the Social Exchange Theory. Together, these perspectives provide a robust explanation for why perfectionist organizational expectations can lead to increased engagement in detrimental behaviors that pose harm to the organization or its employees.

First, the Conservation of Resources Theory [32] states that members actively seek to obtain, keep, and secure resources that they consider valuable. When the resources are threatened, lost, or not adequately restored after investment, individuals experience stress. In the context of OPP, this theory suggests that the constant pressure to meet exceptionally high standards can lead to a continuous expenditure of individual resources including time, energy, and cognitive effort. This ongoing investment would result in a state of resource depletion, leaving employees feeling vulnerable and prone to negative behaviors. Furthermore, perfectionist organizational expectations can pose a constant threat to valued resources such as self-esteem, professional reputation, and job security. The fear of failing to meet these high standards is an ongoing stressor that would result in a state of chronic resource depletion. The intense focus on flawless performance may also leave little opportunity for resource recovery, creating a cycle of resource loss that can lead to exhaustion and frustration. Applying COR theory to our hypothesis, we argue that OPP creates a work environment characterized by continuous resource threat and potential resource loss, which may motivate employees to engage in CWB as a maladaptive coping mechanism or as a form of retaliatory resource conservation behavior [33,34].

Second, the ERI model [35] provides an additional useful perspective on the relationship between OPP and CWB. The model suggests that a lack of reciprocity between high effort and low reward can result in negative emotions, stress reactions, and potentially harmful behaviors. OPP demands sustained high effort from employees to meet exceptionally high standards. However, the extreme nature of these expectations can lead employees to feel that their considerable efforts are not adequately rewarded, either through tangible benefits or intangible recognition. This perceived mismatch between effort expended and rewards received can lead to a state of emotional distress and strain. This paper proposes that OPP may create or exacerbate an effort–reward imbalance, potentially leading to CWB as a means of expressing dissatisfaction or attempting to restore equity [34].

Third, the Social Exchange Theory [36] offers a third lens through which to understand the OPP-CWB relationship. This theory posits that social relationships are based on the reciprocal exchange of resources and that individuals expect a balance in these exchanges. Employees form implicit beliefs about reciprocal obligations between themselves and their organization. OPP would be perceived as a breach of this psychological contract if employees felt that the demands exceeded reasonable expectations. If members feel that their organization’s perfectionist demands exceed the benefits they receive, they may engage in negative reciprocity by withdrawing positive behaviors or engaging in harmful ones. In this context, CWB can be seen as a means of rebalancing in the perceived unfair exchange relationship.

The integration of these theoretical perspectives offers a comprehensive perspective for comprehending how OPP leads to increased CWB. The resource depletion processes highlighted by COR theory, the effort–reward imbalance emphasized by the ERI model, and the negative reciprocity proposed by Social Exchange Theory collectively suggest that perfectionist organizational expectations create conditions conducive to engaging in counterproductive behaviors.

**Hypothesis** **1.** *OPP will increase CWB.*

### 2.2. OPP and Job Insecurity

We propose that OPP may elevate employees’ job insecurity. Job insecurity is a multifaceted construct in organizational psychology and management research, which has received considerable attention due to its prevalence in contemporary work environments. This concept indicates a member’s perception of a lack of control in maintaining desired job stability in the face of potential danger [13]. Existing works have extended the knowledge of the nature and implications of job insecurity. Job insecurity is a personal interpretation that does not always correspond to actual job risks [37]. This subjective nature underscores the importance of individual differences and contextual factors in shaping experiences of job insecurity. The consequences of job insecurity are far-reaching and overwhelmingly negative. Meta-analytic evidence proposes that the concept is related to lower job satisfaction, reduced organizational commitment, and impaired job performance [38,39,40]. It has also been linked to poorer mental and physical health outcomes, such as increased stress, anxiety, and psychosomatic complaints [41,42].

The link between OPP and employee job insecurity can be elucidated through the lens of several complementary theoretical frameworks. This section explores this link through COR theory, Cognitive Appraisal Theory, and Uncertainty Management Theory, providing a multifaceted understanding of how perfectionist organizational expectations may foster perceptions of job insecurity among members.

First, COR theory [32] strengthens the OPP–job insecurity link. In the context of OPP, COR theory offers several insights. First, the constant pressure to meet exceptionally high standards may result in a continuous expenditure of individual resources, including time, energy, and cognitive endeavor. This ongoing investment would result in resource depletion, leaving employees feeling vulnerable and insecure in their work roles. Furthermore, the fear of failing to meet perfectionistic standards poses a constant threat to valued resources such as professional reputation and self-esteem. This ongoing threat would result in a state of chronic stress, which can manifest itself in increased job insecurity. In addition, OPP can create a situation where the resources invested by employees do not yield proportional gains, leading to a negative resource balance [43].

Second, the Cognitive Appraisal Theory [44] provides another valuable perspective on this relationship. This theory suggests that an individual’s response to potential stressors is mediated by their cognitive appraisal of the situation. This theory distinguishes between primary and secondary appraisals, and OPP can affect both primary and secondary appraisals. At the primary appraisal phase, employees may perceive the demand for perfection as a significant threat to their job status. The perceived inability to consistently meet these high standards may be seen as potentially damaging to one’s career prospects. During the secondary appraisal phase, which is related to the assessment of one’s capacity to cope with the perceived threat, OPP may lead employees to doubt their capacity to consistently perform at the required level. This can lead to negative secondary appraisals, further exacerbating feelings of job insecurity [34].

Third, the Uncertainty Management Theory [45] provides additional insights into how OPP might promote job insecurity. This theory suggests that individuals have a fundamental need to manage uncertainty in their environment and that perceptions of fairness play a crucial role in the process. Within the context of OPP, employees may perceive the impossibly high standards as unfair, leading to increased uncertainty about their position in the organization and their ability to meet expectations. The theory suggests that uncertainty in one domain (e.g., ability to meet perfectionistic standards) may increase uncertainty in other domains (e.g., job security). Furthermore, under conditions of uncertainty, individuals may use perceptions of fairness as a heuristic for assessing their value to the organization. Perfectionistic demands may be perceived as unfair, leading to negative self-evaluations and increased job insecurity [33].

The integration of the theoretical frameworks offers a comprehensive basis to understand the potential link between OPP and employee job insecurity. The constant resource drain implied by COR theory, the negative cognitive appraisals suggested by Lazarus and Folkman’s work, and the uncertainty and perceived unfairness highlighted by Uncertainty Management Theory collectively suggest that perfectionist organizational expectations may create an environment conducive to increased job insecurity.

**Hypothesis** **2.** *OPP would increase job insecurity.*

### 2.3. Job Insecurity and CWB

We propose that increased job insecurity among members can significantly augment their risk of CWB. The job insecurity-CWB link is comprehensively explained through the lens of three complementary theoretical frameworks: Social Exchange Theory, Psychological Contract Theory, and Affective Events Theory. These perspectives provide a multifaceted explanation for why perceptions of job insecurity would lead to increased engagement in counterproductive work behaviors.

First, the Social Exchange Theory [36,46] posits that social relationships rely on the reciprocal exchange of resources, both tangible and intangible. Central to this theory is the concept of reciprocity. Employees exchange their time, skills, and effort for various organizational rewards, including job security. If a member feels that his or her job security is under threat, this represents a significant imbalance in this exchange relationship. This perceived imbalance can trigger a process of negative reciprocity in which employees seek to rebalance the exchange by withholding positive behaviors or engaging in negative ones. Job insecurity, as viewed through the prism of this theory, represents a withdrawal of a key organizational offering. In response, employees may participate in CWB as a form of reciprocity, seeking to achieve equity in the reciprocal interaction. This would manifest itself in a variety of ways, from minor acts of deviance to more serious forms of organizational or interpersonal damage [41,47].

Second, the Psychological Contract Theory, introduced by Rousseau [48] and further developed by Morrison and Robinson [49], offers another valuable perspective on the link between job insecurity and CWB. This theory posits that employees form implicit beliefs about reciprocal obligations between themselves and their organization that go beyond the formal employment contract. Job security is a key component of this psychological contract, particularly in traditional employment relationships. When members experience increased levels of job insecurity, they often interpret this as a breach or violation of their psychological contract. Such perceived breaches would profoundly affect employees’ attitudes and behaviors. Violations of the psychological contract are related to several adverse outcomes, including increased CWB. The experience of a breach of contract can evoke feelings of anger, betrayal, and injustice, which may lead to retaliation. Within the realm of job insecurity, members would engage in CWB as a means of expressing dissatisfaction, attempting to regain a sense of autonomy or control over their work situation, or seeking revenge for the perceived violation [39,50].

Third, the Affective Events Theory [51] offers a third theoretical lens for understanding the job insecurity-CWB link. This theory emphasizes the role of workplace events in building members’ emotions, attitudes, and behaviors. According to this theory, job insecurity can be conceptualized as an ongoing negative affective event or a series of events that generate adverse emotional reactions. These emotional reactions, in turn, affect work-related behaviors, including CWB. The theory suggests that the ongoing worry and anxiety associated with job insecurity could deplete employees’ emotional resources, making them more vulnerable to engaging in impulsive or harmful behaviors. Furthermore, Affective Events Theory posits that the cumulative effect of negative affective experiences can lead to changes in work attitudes over time. In the context of job insecurity, this could manifest itself in reduced job satisfaction or organizational commitment, both of which have been linked to increased CWB in previous research [52].

The integration of these three theoretical frameworks provides a comprehensive understanding of the job insecurity-CWB link. Social Exchange Theory explains how job insecurity disrupts the reciprocal employment relationship, potentially triggering negative reciprocity in the form of CWB. Psychological Contract Theory highlights how job insecurity would be considered as a breach of contract, resulting in feelings of violation that may motivate retaliatory behaviors. Affective Events Theory highlights the emotional processes through which job insecurity can lead to CWB, emphasizing the role of negative affect and resource exhaustion.

**Hypothesis** **3.** *Employees’ job insecurity will increase CWB.*

### 2.4. The Mediating Roles of Job Insecurity in the OPP-CWB Link

This research proposes that job insecurity mediates the OPP-CWB link. The Context–Attitudes–Behavior (CAB) framework offers a sound theoretical basis for comprehending the complex relationships between organizational contexts, employee attitudes, and workplace behaviors. This perspective proposes that contextual factors influence individual attitudes, which in turn shape behavioral outcomes [53,54]. Applying this model to our research, we propose that OPP serves as a contextual factor, job insecurity represents an attitudinal response, and CWB emerges as the resulting behavior.

OPP creates a work environment characterized by excessively high standards and expectations [4]. This context would significantly affect members’ perceptions and attitudes towards their job security. When faced with seemingly unattainable performance standards, employees may develop heightened concerns about their ability to meet these expectations, resulting in increased perceptions of job insecurity [55].

Job insecurity, as an attitude, reflects employees’ concerns about the future of their employment [13,38]. The CAB framework suggests that such attitudes mediate the relationship between contextual factors and behavioral outcomes. In this case, job insecurity may serve as a crucial mediating mechanism through which OPP influences CWB.

CWB, which includes activities that cause harm to the organization or its members, can be seen as a behavioral response to feelings of job insecurity [28]. Employees experiencing job insecurity would participate in CWB as a form of coping mechanism or as a way of expressing frustration with their perceived precarious employment situation [38].

**Hypothesis** **4.** *Job insecurity would mediate the OPP-CWB link.*

### 2.5. The Moderating Influence of Self-Efficacy in AI Learning in the OPP–Job Insecurity Link

We posit that self-efficacy in AI learning plays a moderating role, reducing the augmenting influence of OPP on job insecurity. Given our previous discussions, it seems obvious that OPP could increase the degree of job insecurity. However, it is important to recognize that the influence of OPP on job insecurity varies across different organizational contexts.

Self-efficacy in AI learning is an emerging construct that extends the well-established concept of self-efficacy to the domain of AI education and skill acquisition. This particular manifestation of self-efficacy means an individual’s belief in his or her ability to acquire, understand, and use concepts and technologies related to AI [14,15,16,17]. As AI becomes increasingly prevalent in various sectors, understanding the factors that influence individuals’ confidence and motivation to engage in AI learning has gained significant importance in educational and organizational research [16,19,20,21,56]. Self-efficacy in AI learning builds on Bandura’s [18] social cognitive theory and draws parallels with related constructs such as computer self-efficacy [57] and technology self-efficacy [58]. However, it focuses specifically on the unique challenges and complexities associated with AI technologies, such as machine learning, natural language processing, and robotics. In organizational contexts, AI learning self-efficacy has been linked to employees’ willingness to adopt and integrate AI technologies into their work [21,59]. Members with higher AI learning self-efficacy would participate in AI-related training programs and demonstrate openness to AI-driven organizational change [15,16,19]. This emphasizes the potential role of self-efficacy in facilitating the successful implementation of AI technologies in the workplace.

The moderating role of the self-efficacy in the OPP-employee job insecurity link is elucidated by the Social Cognitive Theory [60] and the JD-R model [61]. This theoretical perspective offers a basis for understanding how members’ belief in their capacity to learn and apply AI-related skills may buffer the potentially detrimental effects of perfectionist organizational expectations on perceptions of job security.

The Social Cognitive Theory suggests that self-efficacy plays a critical role in how people approach challenges and threats [18]. In the context of AI learning, self-efficacy represents members’ belief in their capability to acquire and apply AI-related skills and knowledge.

The JD-R model suggests that job demands (such as OPP) would result in distress and negative outcomes, whereas job resources (such as self-efficacy) may buffer these effects [61]. Self-efficacy in AI learning would be seen as a personal resource that can reduce the detrimental influences of perfectionist organizational expectations on job insecurity.

If employees have high levels of the self-efficacy, the impact of OPP on job insecurity would be attenuated. This buffering effect can be attributed to several factors: First, employees with high AI learning self-efficacy may perceive perfectionist standards as opportunities for growth and development rather than threats to their job security. This perspective is consistent with the challenge–hindrance–stressor framework [62], where challenges are seen as potentially beneficial for personal growth and career advancement. Second, high self-efficacy in AI learning would increase proactive behaviors and engagement with AI technologies. Employees with high self-efficacy would engage in innovative work behaviors that can help them meet or exceed perfectionist standards.

For example, in a marketing firm implementing advanced AI-driven customer analytics, an employee with high AI learning self-efficacy may proactively seek out opportunities to integrate AI tools into their workflow. They might view the organization’s perfectionist expectations as a challenge to demonstrate their adaptability and innovative thinking. This employee might engage in self-directed learning, experimenting with new AI applications and consistently improving their performance, thereby reducing their feelings of job insecurity despite high organizational expectations.

Conversely, if employees have low self-efficacy in AI learning, the link between OPP and job insecurity would be strengthened. This exacerbating effect can be explained by the following mechanisms: First, employees with low AI learning self-efficacy may perceive perfectionist demands as overwhelming and unattainable, leading to increased stress and anxiety. This aligns with the cognitive appraisal theory of stress [44], which posits that individuals’ perceptions of their ability to cope with demands significantly influence their stress responses. Second, low self-efficacy in AI learning may lead to avoidance behaviors and resistance to AI adoption. Employees with low levels of the self-efficacy would experience technostress and engage in technology avoidance behaviors, which could further exacerbate their inability to meet perfectionist standards.

For instance, in the same marketing company, an employee with low AI learning self-efficacy may see the implementation of AI-driven analytics and the perfectionist standards it entails as a substantial threat to their job security. They may struggle to engage with AI training programs, avoid using new AI tools, and experience increased anxiety about their performance. This employee may focus on traditional marketing methods, fall behind in meeting the organization’s evolving expectations and increase their job insecurity.

Recent advances in AI efficiency have significant implications for organizational dynamics and employee behavior [63]. For instance, Brougham and Haar [64] propose a new framework for understanding how AI adoption affects job design and employee skill requirements. Their work suggests that AI efficiency can lead to both job enrichment and potential job insecurity, depending on organizational implementation strategies.

**Figure 1:**
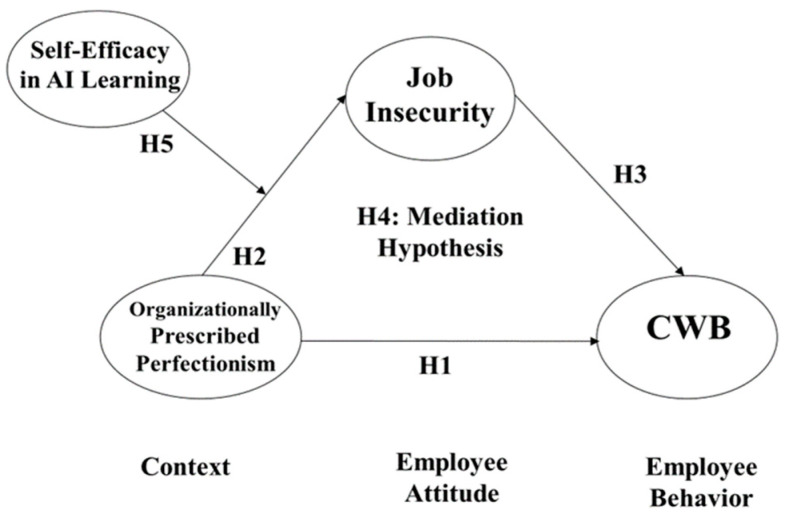
Theoretical model (CWB means counterproductive work behavior).

In terms of self-efficacy in AI learning, recent work by Kim et al. [65] presents a novel theoretical model linking technological self-efficacy to employee adaptability in AI-enhanced work environments. Their research provides a strong foundation for our investigation of self-efficacy as a potential moderator in the relationship between organizationally prescribed perfectionism and job insecurity. The entire research model is depicted in Figure 1.

**Hypothesis** **5.** *Self-efficacy in AI learning would moderate the relationship between OPP and job insecurity, such that high levels of self-efficacy in AI learning would reduce the increasing impact of OPP and job insecurity.*

## 3. Research Methodology

### 3.1. Participants and Procedure

This study included a heterogeneous cohort of professionals, aged 20 and above, hailing from different organizations across South Korea. Our decision to focus on employees aged 20 and above was based on several considerations. First, in the South Korean context, the legal working age is 15, but full-time employment typically begins at 18 or older, often after high school graduation [66]. Second, the focus of our study on OPP and job insecurity required a sample with sufficient exposure to organizational culture and work experience, which we believed would be more prevalent among workers aged 20 and older.

We acknowledge, however, that this decision may have excluded a potentially important segment of the workforce, particularly in relation to self-efficacy in AI learning. Indeed, recent studies have suggested that younger employees, including those under the age of 20, may have higher levels of technological self-efficacy [67]. This limitation of our study opens up important avenues for future research.

Data collection was organized in three separate phases, using a three-wave, time-lagged research design to thoroughly examine changes over time. Participants were recruited through a leading online research platform, known for its extensive database of approximately 5.49 million potential respondents. During the online registration phase, participants confirmed their current employment status and completed a verification step that required either a mobile phone number or an email address to enhance the security of the data collection process. The effectiveness of using digital surveys to collect a broad and diverse sample is well-supported by its demonstrated validity and reliability in previous research [68].

The purpose of this paper was to collect time-lagged data from actively employed individuals within South Korean companies, overcoming the limitations often associated with cross-sectional studies. The advanced digital tools used in this study allowed for accurate tracking of participant engagement throughout the survey period, ensuring consistent participation across all data collection phases. We used a time-lagged design with three waves of data collection, each separated by approximately six weeks, with each survey session remaining open for two to three days to maximize response rates. This approach helps reduce common method biases by temporally separating the measurement of predictor and criterion variables [69]. Specifically, our data collection process took place over a four-month period, from 1 March 2023 to 30 June 2023. The first wave, focusing on OPP and self-efficacy in AI learning, was conducted from 1 March to 15 March 2023. The second wave, measuring job insecurity, took place from 15 April to 29 April 2023. The final wave, assessing counterproductive work behavior, was conducted from 30 May to 13 June 2023. This time-lagged approach allowed us to minimize common method biases [69] and capture potential causal relationships between our variables of interest. The specific timing of our data collection is particularly relevant given the rapid pace of AI adoption in the workplace during this period [70], which may influence our measured constructs, particularly self-efficacy in AI learning. This approach helps reduce common method bias by temporally separating the measurement of predictor and criterion variables [69].

To prevent and detect careless responses, we included attention check items throughout the survey and monitored response times. Responses that failed the attention check or were completed in an inappropriately short time (less than 2 min) were excluded from the analysis. Strict protocols were enforced to maintain data integrity, including the use of geo-IP restriction traps to prevent any overly rapid responses, thus ensuring the quality and credibility of the research findings.

The survey administrators proactively contacted potential participants to invite their participation in the study. The team clearly communicated to potential respondents that their participation was completely voluntary and assured them of the confidentiality and that their data would only be used for research purposes. After obtaining informed consent from willing participants, strict ethical standards were maintained throughout the process. Participants were compensated for their time with a monetary reward of approximately USD 8 or 9.

To minimize the potential for sample bias, the team employed a stratified random sampling technique. This method involved randomly selecting participants from specific strata, which helped to avoid bias associated with demographic and occupational variables, such as gender, age, position, education level, and industry sector. The survey team carefully tracked participant engagement across a range of digital platforms to ensure that the same individuals participated consistently across all three waves of the survey.

To ensure participant confidentiality and ethical data handling, all responses were anonymized and stored on secure, encrypted servers. Participants were informed about the study’s purpose, voluntary nature, and their right to withdraw at any time. The study protocol was approved by the Ethics Committee and IRB of Yonsei University.

In terms of participation, the first phase of the survey included responses from 882 employees. This was reduced to 609 at the second time point, with responses from 415 employees at the final stage. Following the data collection phase, a rigorous cleaning process was conducted to remove any incomplete responses. The final sample size for the study was 412 respondents who completed all phases of the survey, giving a final response rate of 46.71%. The sample size calculation was based on scholarly advice, including the use of G*Power for statistical power analysis.

To determine the appropriate sample size for our study, we performed an a priori power analysis using G*Power 3.1.9.7 [71]. We based our calculations on the most complex analysis in our study, which was the moderated mediation model. For this analysis, we specified the following parameters: test family = F tests, statistical test = linear multiple regression (fixed model, R^2^ increase), α err prob = 0.05, power (1-β err prob) = 0.95, number of predictors tested = 3 (to account for the interaction term in the moderation analysis), and total number of predictors = 7 (including control variables). Based on previous research on organizational behavior [72], we expected a small to medium effect size (f^2^ = 0.10). The G*Power analysis indicated that a minimum sample size of 176 would be required to detect this effect size with 95% power at the 5% significance level. Our final sample of 412 participants, thus, exceeded this minimum requirement, ensuring adequate statistical power for our analyses. The demographic information of participants is described in Table 1.

### 3.2. Measures

In the first phase of the survey, we asked participants about their experience of OPP and their level of self-efficacy in AI learning they received. The second survey focused on assessing their perceptions of job insecurity. In the third survey, we collected data on participants’ levels of CWB. Each of these variables was measured using multi-item scales rated on a 5-point Likert scale. The complete questionnaire used in this study can be found in Appendix A.

#### 3.2.1. OPP (Point in Time 1, as Reported by Employees)

To assess OPP, the current study used six items by adapting the Socially Prescribed Perfectionism subscale of the Multidimensional Perfectionism Scale (MPS) [23] to the organizational context. The original subscale measures the extent to which an individual feels that others have excessive expectations of him or her and judge him or her critically. We modified the items to refer specifically to the perceived perfectionism imposed by one’s organization or workplace. The items include the following: “My organization expects me to succeed at everything I do”, “I find it difficult to meet my organization’s expectations of me”, “My organization readily accepts that I can make mistakes too. (Reverse-scored)”, “My organization expects me to be perfect”, “My organization expects more from me than I am capable of giving”, “People in my organization think I am still competent even if I make a mistake. (Reverse-scored)”. Cronbach’s alpha was reported to be 0.826.

#### 3.2.2. Self-Efficacy in AI Learning (Point in Time 1, as Reported by Employees)

This study used a four-item scale from previous research that adapted Bandura’s self-efficacy scale for use in AI learning [60]. The scale was created in previous research [14]. The following items were specifically analyzed in this study: “I am confident in my ability to learn artificial intelligence technology appropriately in my work”, “I am able to learn artificial intelligence technology to perform my job well, even when the situation is challenging”, “I can develop my competencies needed for my job through AI technology learning”, and “I will be able to learn important information and skills from my AI training”. A Cronbach’s alpha of 0.931 was reported.

#### 3.2.3. Job Insecurity (Time Point 2, as Reported by Employees)

To measure the degree of job insecurity, this study used five items from Kraimer et al.’s [73] scale. The items in this study included: “If my current organization were facing economic problems, my job would be the first to go”, “I will be able to keep my present job as long as I wish (reverse coded)”, “I am confident that I will be able to work for my organization as long as I wish (reverse coded)”, “Regardless of economic conditions, I will have a job at my current organization (reverse coded)”, and “My job is not a secure one”. Cronbach’s alpha was reported to be 0.914.

#### 3.2.4. CWB (Point in Time 3, as Reported by Workers)

At time point 3, CWB was assessed by using five items from the existing CWB scale [74]. The items were “I purposely wasted my employer’s materials/supplies”, “I came to work late without permission”, “I insulted or made fun of someone at work”, “I purposely did my work incorrectly”, and “I took supplies or tools home without permission”. Cronbach’s alpha was reported to be 0.925.

#### 3.2.5. Control Variables

In this study, several control factors were used, including employee tenure, gender, job position, and educational level, to adjust for their possible influences on the dependent variable. These data were collected during the initial survey phase. The rationale for including these specific control variables is based on their established correlation with CWB in the existing literature [75,76], with the aim of minimizing omitted variable bias and enhancing the clarity of the results of our primary variables of interest.

### 3.3. Statistical Analysis

To analyze the relationships between the study variables, we used SPSS version 28 to conduct a Pearson correlation analysis after data collection. We chose structural equation modeling (SEM) as our primary analytical approach because of its ability to simultaneously test complex relationships among multiple variables while accounting for measurement error [77].

In testing our hypotheses, we followed a two-step approach proposed by Anderson and Gerbing [78]: first, we assessed the measurement model; then, we examined the structural model. For the structural analysis, we used the AMOS 26 software and applied the maximum likelihood (ML) estimation method to conduct a moderated mediation analysis. After validating the measurement model using Confirmatory Factor Analysis (CFA), we followed standard structural equation modeling (SEM) procedures.

To assess the adequacy of the model in accurately capturing the empirical data, we used several model fit indices, including the Comparative Fit Index (CFI), the Tucker–Lewis Index (TLI), and the Root Mean Square Error of Approximation (RMSEA). According to established academic standards, model fits are considered satisfactory when the CFI and TLI values are greater than 0.90 and the RMSEA is less than 0.06 [79].

To address potential multicollinearity issues, we calculated variance inflation factors (VIFs) for all predictor variables. All VIF values were less than 3, indicating no significant multicollinearity problems.

## 4. Results

### 4.1. Descriptive Statistics

Our research revealed significant correlations among the key variables of interest: OPP, self-efficacy in learning artificial intelligence, job insecurity, and CWB. These correlations are detailed in Table 2, which is pasted below.

### 4.2. Scale Validation

To ensure the validity and reliability of our measures, we conducted a comprehensive scale validation process. Confirmatory Factor Analysis (CFA) was performed on each scale using AMOS 26. Factor loadings for all items were above the recommended threshold of 0.7, ranging from 0.723 to 0.892. Model fit indices showed a good fit for all scales (CFI > 0.95, TLI > 0.95, RMSEA < 0.06).

Cronbach’s alpha coefficients were calculated to assess internal consistency reliability. All scales showed excellent reliability, with α values ranging from 0.826 to 0.931 [80]. To establish convergent validity, we computed Average Variance Extracted (AVE), and Composite Reliability (CR) values were calculated. AVE values were above the 0.5 threshold (ranging from 0.509 to 0.774), and CR values were above 0.7 (ranging from 0.832 to 0.932), indicating strong convergent validity [81].

To demonstrate discriminant validity, we constructed a correlation matrix with the square root of the AVE on the diagonal. In all cases, the square root of the AVE for each construct was greater than its correlation with other constructs, confirming discriminant validity [77].

### 4.3. Measurement Model

To evaluate the adequacy of our measurement model, we conducted confirmatory factor analysis (CFA) on all items to determine the discriminant validity of our four main variables: OPP, self-efficacy in AI learning, job insecurity, and CWB. We conducted a series of chi-squared difference tests to compare the four-factor model (OPP, self-efficacy in AI learning, job insecurity, and CWB) with alternative configurations. The three-factor model recorded a chi-square value of 1666.997 with 166 degrees of freedom, a CFI of 0.720, a TLI of 0.679, and an RMSEA of 0.148. The two-factor model had a chi-squared value of 2414.788 with 168 degrees of freedom, a CFI of 0.580, a TLI of 0.525, and an RMSEA of 0.180. The single-factor model had a chi-squared value of 3593.121 with 169 degrees of freedom, a CFI of 0.360, a TLI of 0.281, and an RMSEA of 0.222. The fit indices of each model indicated that the four-factor model provided a better fit than the alternatives. The chi-square for the preferred model was 317.755 with 163 degrees of freedom, and it also featured a CFI of 0.971, a TLI of 0.966, and an RMSEA of 0.048. Subsequent chi-squared tests confirmed that the four research variables had sufficient discriminant validity (please see Table 3).

### 4.4. Structural Model

We used a moderated mediation model to explore our hypotheses, integrating both mediation and moderation analyses. Within this framework, we examined how the influence of OPP on CWB was mediated by insecurity. Furthermore, self-efficacy in AI learning was considered as a moderating variable that could potentially attenuate the increasing effect of OPP on job insecurity.

To operationalize our moderation analysis, an interaction term was constructed in the current paper by multiplying the variables representing OPP and self-efficacy in AI learning. To minimize the effects of multicollinearity and maintain the integrity of our correlations, we first centered these variables around their means. This technique not only helped to reduce multicollinearity but also prevented a reduction in correlation strength, thus increasing the reliability of our moderation analysis [82].

To evaluate the degree of potential multicollinearity, we computed variance inflation factors (VIFs) and corresponding tolerance levels, using the methodology outlined by Brace et al. [58]. The results showed that both OPP and self-efficacy in AI learning had VIF values of 1.010, with tolerance indices of 0.990. These metrics indicated that our variables were free from significant multicollinearity concerns, as the VIF values were well below the commonly accepted threshold of 10, and the tolerance levels were well above the minimum criterion of 0.2 [82].

#### 4.4.1. Findings from the Mediation Evaluation

To determine the optimal mediation model, we conducted a chi-squared difference test to assess whether a full mediation model was more appropriate than a partial mediation model. The full mediation model excluded the direct link between OPP and CWB, in contrast to the partial mediation model. Both models showed good fit; the full mediation model showed fit indices of (χ^2^ = 378.498 (df = 195), CFI = 0.956, TLI = 0.948, RMSEA = 0.048), whereas the partial mediation model showed (χ^2^ = 356.004 (df = 194), CFI = 0.961, TLI = 0.954, RMSEA = 0.045). However, the results of the chi-squared difference test suggested that the partial mediation model provided a better fit, as indicated by the chi-squared difference (Δχ^2^ [1] = 22.494, *p* < 0.01) between the two models. This result suggests that OPP affects CWB both directly and indirectly, through job insecurity. In addition, we used control variables, such as tenure, gender, education level, and occupational position. These variables, however, did not reach statistical significance in influencing CWB.

Our analysis, incorporating these control variables, revealed a significant direct link from OPP to CWB in the partial mediation model (β = 0.262, *p* < 0.001), supporting Hypothesis 1. This finding underscores the adequacy of the partial mediation model, which ultimately emerged as the preferred model due to its significant direct pathway from OPP to CWB and superior fit indices. This comparison confirms the relevance of the partial mediation model and illustrates that the influence of OPP on CWB is mediated by job insecurity.

Our findings also support Hypothesis 2, which indicates a strong positive effect of OPP on job insecurity (β = 0.235, *p* < 0.001), and Hypothesis 3, which shows that job insecurity significantly increases CWB (β = 0.195, *p* < 0.001). The results are presented in Table 4.

#### 4.4.2. Bootstrapping

In order to test Hypothesis 4, which suggests that the link between OPP and CWB is mediated by job insecurity, the present study utilized a bootstrapping technique with a substantial sample size of 10,000, as recommended by Shrout and Bolger [83]. In order to establish the statistical significance of the indirect effect of OPP on CWB, the 95% bias-corrected confidence interval (CI) should not include zero. In our robust analysis, the bootstrapping results indicated a 95% CI ranging from 0.018 to 0.091 for the indirect path, clearly excluding zero. This statistically significant result supports the mediating effect of job insecurity and highlights its importance in linking OPP to CWB. These findings are presented in Table 5.

#### 4.4.3. Findings from the Moderation Evaluation

**Figure 2:**
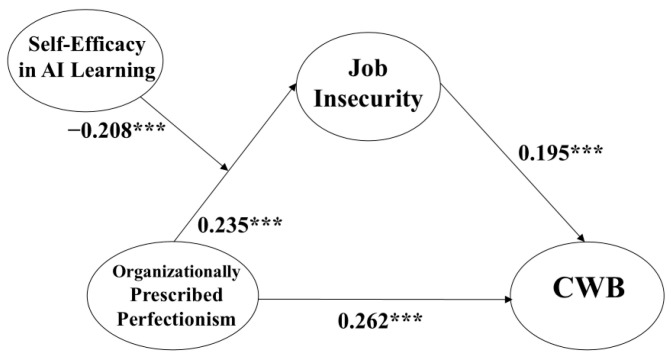
Coefficient values of our research model (*** *p* < 0.001. All values are standardized, CWB means counterproductive work behavior).

**Figure 3:**
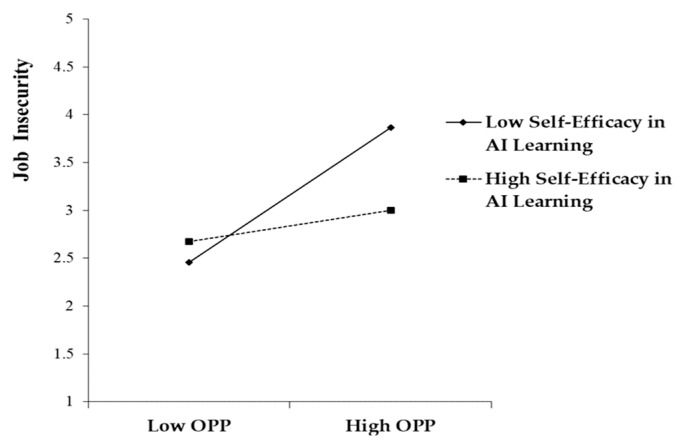
Moderating effect of self-efficacy in AI learning in the OPP–job insecurity link.

The focus of our moderation analysis was to explore the possible moderating effect of self-efficacy in AI learning on the link between OPP and job insecurity. To achieve this, an interaction term was constructed by adjusting the means of OPP and the self-efficacy, which helped to manage potential multicollinearity and maintain the clarity of our analysis. The interaction term was statistically significant (β = −0.208, *p* < 0.001), suggesting that self-efficacy in AI learning indeed plays a crucial role in attenuating the increasing effect of OPP on job insecurity. The significant result provides robust support for Hypothesis 5, confirming that self-efficacy would effectively reduce the detrimental effects of high OPP (please see Figure 2 and Figure 3).

## 5. Discussion

Our results provide essential understanding of the complex interplay between OPP, job insecurity, CWB, and self-efficacy in learning AI in the modern organizational context. Our results not only support the hypothesized relationships but also extend our understanding of how perfectionist organizational cultures may influence employee behavior in an era of rapid technological progress.

First, our study confirms a direct positive relationship between OPP and CWB, which is consistent with and extends previous research on individual-level perfectionism and its negative consequences [8]. This finding aligns with and extends the COR theory [32]. Whereas COR theory posits that individuals strive to obtain and protect valued resources, our findings suggest that OPP may pose a persistent threat to these resources, leading to CWB as a maladaptive coping mechanism. This extends the application of COR theory to the domain of organizational perfectionism, providing new insights into how perfectionist cultures may deplete employee resources.

However, our findings differ somewhat from the challenge–hindrance stressor framework [62]. While this framework suggests that some forms of stress can be motivating, our findings indicate that the stress induced by OPP is predominantly a hindrance and consistently leads to negative outcomes. This difference highlights the unique nature of perfectionist stressors in organizational settings.

Our findings on the relationship between OPP and CWB align with recent international research. For instance, Gillet et al. [84] conducted a study in French and Canadian organizations and found that perfectionist work cultures are associated with increased employee burnout and deviance, particularly in high-pressure industries.

Second, our results show that OPP is positively associated with job insecurity, which in turn results in increased CWB. This mediation effect provides a nuanced understanding of the psychological processes through which perfectionist organizational cultures may promote negative employee behaviors. The finding is consistent with existing works on job insecurity as a significant workplace stressor [13] and extends this line of inquiry by specifically linking it to organizational perfectionism. This relationship can be interpreted through Uncertainty Management Theory, where the constant pressure to meet impossibly high standards may create a sense of uncertainty about one’s job stability, leading to increased job insecurity.

The mediating role of job insecurity in the OPP-CWB link underscores the significance of considering employees’ perceptions of job stability when investigating the effects of high-pressure organizational cultures. This finding suggests that perfectionist expectations may not only influence CWB directly but also indirectly affect it by creating an environment of perceived instability and threat to one’s job.

Our findings on the mediating role of job insecurity in the OPP-CWB relationship are supported by recent international research. A cross-cultural study by Moy et al. [85] across 35 countries (27 members of the European Union, and the United Kingdom, Albania, Macedonia, Montenegro, Serbia, Turkey, Switzerland, and Norway) found that perceived job insecurity consistently mediated the relationship between various organizational stressors and negative work outcomes. Our study extends these findings by specifically identifying OPP as a significant antecedent of job insecurity across cultures.

Third, a particularly innovative aspect of our study is the recognition of self-efficacy in AI learning as a critical moderating factor in the dynamic between OPP and job insecurity. The reducing impact of AI learning self-efficacy on the dynamic suggests that a member who is confident in his or her capacity to understand and adopt AI technologies is less likely to experience job insecurity under perfectionist organizational pressures. This observation is consistent with Social Cognitive Theory [60] and extends its relevance to adapting to technology in demanding work environments.

The role of self-efficacy in AI learning underscores the importance of technological literacy and adaptability in modern workplaces. As organizations increasingly adopt AI and other sophisticated technologies, an employee’s belief in his or her capacity to master and utilize these technologies would serve as a safeguard against the detrimental influences of perfectionist organizational cultures. This finding has important implications for both theoretical frameworks and practical applications, suggesting that enhancing technological self-efficacy may be an important approach in mitigating the adverse effects associated with high-performance demands.

Regarding the moderating effect of self-efficacy in AI learning, our findings contribute to the growing body of international literature on technological adaptation in the workplace [15,16,17]. Interestingly, our findings on the moderating effect of self-efficacy in AI learning are consistent with recent work on self-efficacy in AI. For example, Kim and Kim [14] showed that self-efficacy in AI learning mitigates the detrimental effects of work overload on psychological contract violation. Kim et al. [65] also showed that employee self-efficacy in AI use moderates the relationship between job insecurity and psychological safety. This similarity highlights the importance of considering AI-specific self-efficacy in increasingly technology-driven work environments.

In conclusion, our study both corroborates and extends existing theoretical frameworks and empirical findings. The positive relationship between OPP and CWB aligns with broader stress theories but offers new insights into the specific effects of perfectionist organizational cultures. The mediating role of job insecurity is consistent with international findings, suggesting a potentially universal mechanism linking organizational stressors to negative outcomes. However, our findings on the moderating role of AI learning self-efficacy introduce a novel perspective, highlighting the importance of technology-specific psychological resources in contemporary work environments.

### 5.1. Theoretical Implications

This study offers several important theoretical implications that address critical gaps in the existing literature.

First, our study expands the concept of socially prescribed perfectionism to the organizational context by introducing and empirically testing the construct of OPP. This novel perspective enriches our understanding of how perfectionist cultures manifest and operate within modern business environments. By applying the well-established concept of socially prescribed perfectionism [23] to the organizational level, we fill a significant gap in the literature, which has predominantly focused on individual-level perfectionism. Our findings suggest that OPP operates as a distinct construct with unique antecedents and consequences in the workplace. This extension of perfectionism theory to the organizational level opens up new avenues for research on the interplay between organizational culture, employee perceptions, and workplace behaviors. Future studies could explore how OPP differs from other forms of organizational pressure and how it interacts with individual differences in shaping employee outcomes.

Second, this research sheds light on the mediating processes through which OPP affects the behavior of members, specifically CWB, by examining job insecurity as a mediating factor. This addresses a critical research gap within the published work on the processes that link organizational expectations to employee behaviors. By integrating Uncertainty Management Theory with perfectionism research, we provide a more nuanced understanding of how perfectionist organizational cultures can create feelings of uncertainty and insecurity among employees, ultimately leading to negative behaviors. This theoretical integration offers a new prism through which to examine the psychological influence of high-pressure working environments. Future work should build on this framework to identify other potential mediators in the link between OPP and employee outcomes, including psychological contract violation or perceived organizational support.

Third, our research adds to the growing body of literature on the impact of personal resources in reducing negative outcomes at work. Specifically, we delve into how self-efficacy in AI learning influences the link as a moderator. This addresses a significant gap in understanding how individual differences in technological adaptability would reduce the detrimental effects of perfectionist organizational cultures. By drawing on Social Cognitive Theory [60] and applying it to the realm of AI at work, we extend the applicability of the theory to contemporary work environments characterized by rapid technological change. The results of this study suggest that personal resources related to technological adaptation play a crucial role in how members respond to high organizational expectations in increasingly digitalized workplaces. This opens up new theoretical avenues for exploring the intersection of perfectionism, technology adoption, and employee well-being in the digital age.

Lastly, by incorporating these elements into a comprehensive model, our research provides a holistic framework for understanding the complex interplay between organizational expectations, employee perceptions, and behavioral consequences in the context of technological change. This integrated approach addresses the gap in the literature, which has often examined these factors in isolation. By synthesizing perspectives from perfectionism theory, job insecurity research, and technology adaptation studies, we offer a more complete picture of the modern workplace dynamics. This comprehensive model demonstrates the value of multi-theoretical approaches in organizational research and suggests that future work must consider exploring the intersections between organizational culture, employee perceptions, and technological change. Such integrated approaches can offer a more nuanced knowledge of employee behavior in increasingly complex and demanding work environments.

Together, these theoretical implications advance our understanding of the unintended consequences of organizational practices aimed at achieving excellence, the psychological processes that mediate the influences of these practices on the behavior of members, and the role of individual differences in technological adaptation in moderating these effects. They call for a more holistic approach to the study of organizational behavior that takes into account the complex interactions between organizational culture, employee perceptions, and technological change. Future research building on these implications may lead to the development of more sophisticated theories that can better explain and predict employee behavior in the dynamic and ever-changing environment of modern organizations.

### 5.2. Practical Implications

The current research has several significant practical implications for top management teams, leaders, and practitioners, addressing critical gaps in organizational practice and providing actionable insights for fostering healthy, productive work environments in the face of increasing technological advances and competitive pressures.

First, our research highlights the critical need for organizations to re-evaluate and calibrate their performance expectations in light of the potential negative consequences of OPP. Top management teams need to recognize that while the pursuit of excellence is commendable, extreme perfectionist standards can lead to CWB and ultimately undermine organizational effectiveness. To address this, leaders should adopt a more balanced approach to performance management that emphasizes continuous improvement rather than flawless execution. For instance, organizations could adopt the ‘Kaizen’ philosophy of incremental improvement [86], which focuses on sustained progress rather than immediate perfection. This approach could include setting challenging but achievable goals, celebrating incremental successes, and fostering a culture that views mistakes as learning opportunities rather than failures. In this way, organizations can maintain high standards while reducing the pressures that can lead to job insecurity and CWB.

Second, our findings underscore the importance of addressing employees’ job insecurity as a key factor in the link between OPP and CWB. Practitioners should develop comprehensive strategies to enhance employees’ perceptions of job security, even in highly demanding work environments. This could include establishing transparent communication channels about organizational performance and individual contributions, providing clear career development paths and offering skill development programs that are aligned with evolving organizational needs. For example, companies could draw inspiration from IBM’s “New Collar” jobs initiative, which focuses on skills rather than degrees, and provides continuous learning opportunities for employees [87]. By proactively addressing concerns about job insecurity, organizations can reduce the detrimental effects of high-performance expectations on employee behavior.

Third, our research emphasizes the critical role of self-efficacy in AI learning as a potential buffer against the negative influences of OPP. In light of this, organizations should prioritize the development of AI-related skills and confidence among their workforces. This could include creating comprehensive AI training programs, establishing mentorship systems for AI skills development, and providing ample opportunities for hands-on experience with AI technologies. Companies could consider adopting approaches similar to Google’s “AI for everyone” initiative, which aims to democratize AI knowledge across the organization [88]. By increasing employees’ self-efficacy, organizations can not only reduce the potential negative effects of high-performance expectations but also better position themselves for success in an increasingly AI-driven business landscape.

Lastly, this research underscores the need to embrace a holistic strategy to effectively manage the culture within an organization in the context of rapid technological change and intense competition. Practitioners should seek to create a balanced organizational culture that rewards high performance while prioritizing employee well-being and adaptability. This could include conducting regular cultural assessments to identify and address potential sources of excessive pressure or insecurity. Organizations could consider adopting elements of agile methodologies beyond software development, emphasizing flexibility, continuous feedback, and iterative progress [89]. Furthermore, leaders should focus on developing emotional intelligence and adaptive leadership skills to better deal with the complexities of managing high-performance cultures in technologically advanced environments. By fostering a more adaptive and employee-centered organizational culture, companies can maintain their competitive edge while minimizing the risk of counterproductive behaviors.

These practical implications collectively call for a more nuanced and holistic approach to managing high-performance cultures in the age of AI and intense global competition. They suggest that organizations can maintain their pursuit of excellence while simultaneously creating work environments that support employee well-being, encourage technological adaptation, and discourage counterproductive behaviors. By implementing these recommendations, top management teams and leaders can create more sustainable, adaptable, and employee-friendly high-performance cultures that drive long-term organizational success.

### 5.3. Limitations and Suggestions for Future Studies

Although the current study provides strong perspectives on the associations among OPP, job insecurity, CWB, and self-efficacy in AI learning, several limitations should be acknowledged, each of which provides avenues for future research.

First, our study was conducted exclusively in South Korea, which may make it difficult to apply our findings to other cultural settings. Organizational cultures and perceptions of perfectionism may vary significantly across different countries and cultures [90]. Future works should aim to duplicate the results of this study in a variety of cultural contexts to delve into the cross-cultural validity of our findings. In addition, cross-national comparative studies may shed light on how cultural factors may moderate the relationships observed in our study, as suggested by recent cross-cultural research on perfectionism [91].

Second, although our sample included professionals from various corporations, we did not specifically analyze industry-specific effects. Different industries may have different norms and expectations regarding perfectionism and AI adoption, which could affect the relationships in our model [92]. Future studies should consider industry as a potential moderating factor and explore how the dynamics of OPP, job insecurity, and CWB may differ across industries, such as technology, finance, healthcare, or manufacturing.

Third, although our three-wave time-lagged design provides some insights into causal relationships, it does not capture long-term effects or potential reciprocal relationships among our variables. Future research could benefit from extended longitudinal designs that track changes over longer time periods, as recommended by Ployhart and Vandenberg [93]. Such designs could reveal how the relationships between OPP, job insecurity, and CWB evolve over time and explore potential feedback loops, as suggested by recent organizational behavior research [94].

Fourth, while our study focused on job insecurity as a mediator and self-efficacy in AI learning as a moderator, other potential mediating and moderating variables warrant investigation. Future research could explore additional mediators such as job involvement, organizational commitment, or psychological contract violation [95]. Similarly, other potential moderators such as emotional intelligence, resilience, or organizational support could be examined, as suggested by recent studies on workplace stressors [96].

Fifth, this study is based on self-reported measures, which would be subject to common methodological biases and social desirability effects, particularly for sensitive constructs like CWB [69]. Future works might benefit from incorporating data from multiple sources, including supervisor ratings of CWB or objective measures of job performance. In addition, experimental or quasi-experimental designs could offer stronger evidence of causal links, as recommended by Antonakis et al. [97].

Sixth, while our study considered self-efficacy in AI learning, we did not delve deeply into specific AI technologies or applications. Future research could investigate how different types of AI technologies might differentially affect the relationships in our model, as suggested by recent studies of AI adoption [98]. Moreover, studies could explore how the pace of technological change within an organization moderates the effects of OPP on employee outcomes, building on recent work on technological change and employee well-being [99].

Seventh, a notable limitation of our study is the exclusion of employees under the age of 20. As pointed out in recent research (e.g., Ma and Fang [100]), younger employees, particularly those in the Generation Z cohort, may have different characteristics in terms of technological adaptability and AI learning self-efficacy. Future studies should consider expanding the age range to include younger workers, which may capture important generational differences in the relationships we have examined. Such research could provide valuable insights into how age and generational factors moderate the effects of organizationally prescribed perfectionism on job insecurity and counterproductive work behavior, particularly in the context of AI adoption.

Eighth, it is important to note that our data collection occurred during a period of significant technological change and economic uncertainty [52]. These contextual factors may have influenced our participants’ responses, particularly with regard to job insecurity and AI learning self-efficacy. Future research may benefit from longitudinal designs that capture these constructs over longer periods of time, potentially revealing how they evolve in response to changing technological and economic landscapes.

Ninth, although our sample size (*N* = 412) exceeded the minimum requirement determined by our G*Power analysis, it is worth noting that larger sample sizes can provide even greater statistical power and precision in estimating effect sizes [101]. Future studies could consider using larger sample sizes to further increase the robustness of findings in this area of research.

Future research should explore how the relationships observed in our study may differ across various age groups, with a particular focus on very young employees (under 20). Such studies could employ comparative designs to examine potential generational differences in AI learning self-efficacy and its moderating effects on organizationally prescribed perfectionism and job insecurity.

Addressing these limitations in future work will not only facilitate the knowledge of the complexities surrounding organizationally prescribed perfectionism but also provide more robust and actionable insights for practitioners.

## 6. Conclusions

This study provides a comprehensive examination of the intricate relationships between OPP, job insecurity, CWB, and self-efficacy in AI learning within the contemporary organizational landscape. By integrating these constructs into a cohesive model, the current research makes significant contributions to the fields of organizational behavior, perfectionism studies, and the emerging field of AI in the workplace.

Our findings extend the concept of socially prescribed perfectionism to the organizational level, introducing OPP as a distinct construct with significant implications for employee behavior and well-being. The direct positive link from OPP to CWB underscores the potential negative consequences of fostering overly high standards within organizational cultures. This relationship, mediated by job insecurity, highlights the complex psychological processes through which perfectionist expectations can lead to detrimental employee behaviors.

Our research highlights the important role of job insecurity as a mediating mechanism between OPP and CWB. This finding not only adds to the growing body of literature on job insecurity as a workplace stressor but also provides new insights into how organizational cultures characterized by perfectionism can generate feelings of insecurity among employees. The identification of this mediating pathway offers valuable implications for organizational interventions aimed at reducing CWB and enhancing employee well-being.

The moderating effect of self-efficacy in learning AI in the OPP–job insecurity link represents a novel contribution to the previous work. This finding bridges the gap between perfectionism studies and research on technological adaptation in the workplace. It suggests that fostering a member’s confidence in his or her capacity to learn and adapt to AI technologies may act as a buffer against the detrimental influences of perfectionist organizational cultures, particularly in terms of job insecurity.

While this study provides valuable insights into the relationships among organizationally prescribed perfectionism, job insecurity, counterproductive work behaviors, and self-efficacy in AI learning, several limitations should be acknowledged. First, our data were collected from a single cultural context (South Korea), which may limit the generalizability of our findings to other cultural settings [102]. Second, despite our time-lagged design, the possibility of reverse causality cannot be completely ruled out [103]. Third, our reliance on self-report measures may have introduced a common method bias, although we took steps to minimize this issue through our research design [104].

Building on our findings and acknowledging our limitations, we suggest several avenues for future research:

1. Cross-cultural studies: Future research should examine the relationships identified in our study across different cultural contexts to assess the generalizability of our findings [105].
2. Longitudinal designs: The use of true longitudinal designs would provide stronger evidence for causal relationships and allow for the examination of how these relationships evolve over time [93].
3. Multi-source data: Future studies could benefit from incorporating supervisor or peer ratings of counterproductive work behavior to address potential common method bias concerns [69].
4. Exploration of additional moderators: Researchers could examine other potential moderators, such as organizational support or individual personality traits, to further understand the conditions under which organizationally prescribed perfectionism leads to negative outcomes [106].
5. Examination of positive outcomes: While our study focused on negative outcomes, future research could explore potential positive outcomes of organizationally prescribed perfectionism, such as increased innovation or job performance, under certain conditions [107].

These future directions would contribute to a more comprehensive understanding of the complex dynamics surrounding perfectionism in organizational settings and its implications for employee behavior and well-being in the context of technological advancements.

In conclusion, the current study significantly advances our understanding of the interplay between organizational perfectionism, employee perceptions, and behaviors in the context of technological advancement. By illuminating the role of job insecurity and AI learning self-efficacy, our research provides a nuanced perspective on the challenges and opportunities facing modern organizations as they navigate the demands of excellence in an increasingly AI-driven world. As organizations continue to grapple with the pressures of perfectionism and technological change, our findings offer valuable insights for fostering healthier, more adaptable workplace cultures that balance high performance with employee well-being.

## Figures and Tables

**Table 1:** Descriptive characteristics of the sample.

Characteristic	Percent
**Gender**	
Men	51.7%
Women	48.3%
**Age (years)**	
20–29	20.9%
30–39	23.3%
40–49	28.2%
50–59	27.7%
**Education**	
High school or below	12.1%
Community college	18.4%
Bachelor’s degree	56.6%
Master’s degree or higher	12.9%
**Position**	
Staff	41.0%
Assistant Manager	16.7%
Manager or deputy general manager	24.5%
Department/general manager or director and above	17.7%
**Firm Size**	
1–9 employees	14.8%
10–29 employees	18.0%
30–49 employees	10.4%
50–99 employees	16.0%
100–149 employees	8.5%
150–299 employees	6.8%
300–449 employees	4.4%
500–999 employees	6.6%
1000–4999 employees	8.7%
Above 5000 employees	5.8%
**Industry Type**	
Manufacturing	22.8%
Services	16.4%
Construction	11.4%
Health and welfare	17.0%
Information service and telecommunications	8.5%
Education	16.5%
Financial/insurance	2.4%
Consulting and advertising Others	0.7%
Others	6.6%

**Table 2:** Correlation among research variables.

	Mean	S.D.	1	2	3	4	5	AVE	CR
1. Tenure_T1	66.70	71.91	−	0.713	-				
2. OPP_T1	2.94	0.69	−0.14	-	0.879			0.509	0.832
3. SE_T1	3.37	0.84	0.06	−0.10 *	-	0.825		0.774	0.932
4. JI_T2	2.81	0.93	−0.04	0.20 **	−0.11 *	-	0.844	0.681	0.914
5. CWB_T3	2.20	0.89	−0.02	0.32 **	−0.05	0.24 **	- *	0.713	0.925

Notes: * *p* < 0.05. ** *p* < 0.01. S.D. indicates standard deviation, OPP indicates organizationally prescribed perfectionism, SE indicates self-efficacy in AI Learning, JI indicates job insecurity, and CWB indicates counterproductive work behavior. For gender, males are coded as 1 and females as 2. For position, general manager or higher is coded as 5, deputy general manager and department manager 4, assistant manager 3, clerk 2, and others below clerk as 1. For education, “below high school diploma” level is coded as 1, “community college” level as 2, “bachelor’s” level as 3, and “master’s degree or more” level is coded as 5. AVE means average variance extracted, and CR means composite reliability. This table includes the square root of the AVE for each construct along the diagonal and the correlations between constructs below the diagonal to support discriminant validity.

**Table 3:** Results of measurement model.

Model	χ^2^	df	CFI	TLI	RMSEA	Comparison	Adaptation
4-factor	317.755	163	0.971	0.966	0.048	4-factor vs. 3-factor	4-factor
3-factor	1666.997	166	0.720	0.679	0.148	3-factor vs. 2-factor	4-factor
2-factor	2414.788	168	0.580	0.525	0.180	2-factor vs. 1-factor	4-factor
1-factor	3593.121	169	0.360	0.281	0.222		

Notes: CFI = Comparative Fit Index; TLI = Tucker–Lewis Index; RMSEA = Root Mean Square Error of Approximation.

**Table 4:** Results of the structural model.

Hypothesis	Path (Relationship)	Unstandardized Estimate	S.E.	Standardized Estimate	Supported
1	OPP → CWB	0.455	0.105	0.262 ***	Yes
2	OPP → Job Insecurity	0.432	0.104	0.235 ***	Yes
4	Job Insecurity → CWB	0.185	0.050	0.195 ***	Yes
5	OPP × Self-Efficacy in AI Learning	−0.270	0.064	−0.208 ***	Yes

Notes: *** *p* < 0.05. Estimate indicates standardized coefficients. S.E. means standard error. OPP means organizationally prescribed perfectionism. CWB means counterproductive work behavior.

**Table 5:** Direct, indirect, and total effects of the final research model.

Model (Hypothesis 4)	Direct Effect	Indirect Effect	Total Effect
OPP → Job Insecurity → CWB	0.262	0.046	0.308

Notes: All values are standardized. OPP means organizationally prescribed perfectionism, CWB means counterproductive work behavior.

## Data Availability

New data were created and analyzed in this study. Data sharing is not applicable to this article.

## Appendix A. Measures

1. OPP (Time Point 1, collected from employees)
  (a) “My organization expects me to succeed at everything I do”.
  (b) “I find it difficult to meet my organization’s expectations of me”.
  (c) “My organization readily accepts that I can make mistakes too”.
  (d) “My organization expects me to be perfect”.
  (e) “My organization expects more from me than I am capable of giving”.
  (f) “People in my organization think I am still competent even if I make a mistake”.
2. Self-Efficacy in AI Learning (Time point 1, collected from employees)
  (a) “I am confident in my ability to learn artificial intelligence technology appropriately in my work”.
  (b) “I am able to learn artificial intelligence technology to perform my job well, even when the situation is challenging”.
  (c) “I can develop my competencies needed for my job through AI technology learning”.
  (d) “I will be able to learn important information and skills from my AI training”.
3. Job Insecurity (Time point 2, collected from employees)
  (a) “If my current organization were facing economic problems, my job would be the first to go”.
  (b) “I will be able to keep my present job as long as I wish”.
  (c) “I am confident that I will be able to work for my organization as long as I wish”.
  (d) “Regardless of economic conditions, I will have a job at my current organization”.
  (e) “My job is not a secure one”.
4. Counterproductive Work Behavior (Time point 3, collected from immediate leader)
  (a) “I purposely wasted my employer’s materials/supplies”.
  (b) “I came to work late without permission”.
  (c) “I insulted or made fun of someone at work”.
  (d) “I purposely did my work incorrectly”.
  (e) “I took supplies or tools home without permission”.
